# Alienist and Neurologist

**Published:** 1881-12-20

**Authors:** 


					﻿EDITORIALS AND MISCELLANEOUS.
ALIENIST AND NEUROLOGIST
Is a quarterly journal of Scientific, Clinical and Forensic Psych-
iatry and Neurology, intended especially to subserve the wants of
the general practitioner of medicine; edited by C. H. Hughes, M.
D., and an associate corps of co-laborers: Terms, $5.00 per annum
in advance. Published in St. Louis, Missouri.
The work embraces- a difficult but interesting department of
Medical Science. It is ably edited, and should be read by every
scientific and progressive medical man.
				

